# How do bacteraemic patients present to the emergency department and what is the diagnostic validity of the clinical parameters; temperature, C-reactive protein and systemic inflammatory response syndrome?

**DOI:** 10.1186/1757-7241-22-39

**Published:** 2014-07-15

**Authors:** Katrine Prier Lindvig, Daniel Pilsgaard Henriksen, Stig Lønberg Nielsen, Thøger Gorm Jensen, Hans Jørn Kolmos, Court Pedersen, Pernille Just Vinholt, Annmarie Touborg Lassen

**Affiliations:** 1 Department of Emergency Medicine, Odense University Hospital, Sdr. Boulevard 29, 5000 Odense C, Denmark; 2 Department of Infectious Diseases, Odense University Hospital, Sdr. Boulevard 29, 5000 Odense C, Denmark; 3 Department of Clinical Microbiology, Odense University Hospital, Sdr. Boulevard 29, 5000 Odense C, Denmark; 4 Department of Clinical Biochemistry and Pharmacology, Odense University Hospital, Sdr. Boulevard 29, 5000 Odense C, Denmark

**Keywords:** Bacteraemia, Emergency medicine, C-reactive protein, Temperature, Systemic inflammatory response syndrome

## Abstract

**Objective:**

Although blood cultures are often ordered based on the presence of fever, it is a clinical challenge to identify patients eligible for blood cultures. Our aim was to evaluate the diagnostic value of temperature, C-reactive-protein (CRP), and Systemic Inflammatory Response Syndrome (SIRS) to identify bacteraemic patients in the Medical Emergency Department (MED).

**Methods:**

A population-based cohort study including all adult patients at the MED at Odense University Hospital between August 1st 2009 - August 31st 2011.

**Results:**

11,988 patients were admitted to the MED within the study period. Blood cultures were performed on 5,499 (45.9%) patients within 2 days of arrival, of which 418 (7.6%) patients were diagnosed with bacteraemia. This corresponded to 3.5% of all patients. 34.1% of the bacteraemic patients had a normal rectal temperature (36.0°–38.0°C) recorded at arrival, 32.6% had a CRP < 100 mg/L and 28.0% did not fulfil the SIRS criteria.

For a temperature cut-point of >38.0°C sensitivity was 0.64 (95% CI 0.59–0.69) and specificity was 0.81 (0.80–0.82) to identify bacteraemic patients.

**Conclusion:**

One third of the acute medical bacteraemic patients had a normal temperature at arrival to the MED. A normal temperature combined with a CRP < 100 mg/L and no SIRS criteria, ruled out bacteraemia.

## Introduction

Early identification of patients with bacteraemia in the Medical Emergency Department (MED) is a daily clinical challenge, from both a resource and clinical perspective. Untreated bacteraemia may lead to sepsis, severe sepsis or septic shock with an associated mortality of up to 30%–50% [1]. Early appropriate antibiotic treatment and fluid resuscitation reduces mortality in septic patients [2,3]. Therefore, early diagnosis and treatment of bacteraemic patients is important [4]. In the MED there is a low positivity rate for blood cultures of 4–8%, however, obtaining too few blood cultures might increase the risk of overlooking bacteraemia and could be fatal as the patient might be withheld correct antibiotic and other disease specific treatments [1]

Published guidelines do not clearly state when blood cultures should be drawn but previous studies of emergency department patients have suggested body temperature, C-reactive-protein (CRP) and Systemic Inflammatory Response Syndrome (SIRS) as predictors of bacteraemia [5-11]. However, the value of these as clinical predictors in the MED needs further clarification. Previous studies have developed more complex models for predicting bacteraemia and some found good performance, but are until now not validated in other populations [1].

The aim of this study was to evaluate the diagnostic performance of the clinical parameters; temperature, CRP and SIRS as predictors of community-acquired bacteraemia in patients admitted to the MED.

## Methods

Study design and participants

We conducted a population-based cohort study consecutively enrolling all first-time admissions among adult patients (age >15 years) arriving at the MED at Odense University Hospital, between August 1st 2009 - August 31st 2011. The hospital is an 1100 bed level 1-trauma centre and a university teaching hospital with all specialities present. It serves both as a tertiary and primary hospital with a primary catchment area of 288,000 persons. The MED had approximately 9,000 admissions annually during the observation period.

### Data sources

All patients had their blood pressure, pulse rate, respiratory frequency, oxygen saturation, rectal temperature, and level of consciousness/Glasgow Coma Scale measured or calculated at arrival, and had standard blood samples drawn including leukocyte count, platelet count, CRP, creatinine, PT-INR and bilirubin. Additional to these standard diagnostic tools, a proportion of patients had an arterial blood test done including PaO_2_, lactate and pH. The decision to draw blood cultures and arterial blood tests was made by the attending physician, and patients without blood cultures, were classified as negative/non-bacteraemic. We linked all included patients to the hospital’s biochemistry and microbiology registries, as well as the Danish National Patient Register [12] and the Danish Civil Registration Register to describe microbiological and biochemical results as well as comorbid conditions. Linkage between the databases used in this study was possible using the Danish civil registration number, a unique personal identification number assigned to every Danish citizen at birth [13].

### Microbiological methods

The blood cultures were incubated and screened for growth of microorganisms for 6 days or until detected positive, using the Bactec 9240 system (Becton Dickinson, NJ, USA) until January 2011 and the Bact/Alert system (BioMérieux) thereafter. Routine methods for identification of bacteria were based on conventional characterisation [14], the Danish reference programme (http://www.dskm.dk), and automated identification using Vitek 2 (bioMérieux) and MALDI-TOF (SARAMIS, bioMérieux).

### Definitions

Community-acquired bacteraemia was defined as having a positive blood culture drawn within the first two days of admission. A blood culture consisted of two blood culture sets, each comprising one aerobic and one anaerobic bottle, and we defined bacteraemia as either: [1] recognised pathogens detected in ≥1 blood culture, or [2] common skin contaminants (coagulase-negative staphylococci, *Bacillus* spp, *Propionibacterium* spp, *Corynebacterium* spp, viridans group streptococci, *Aerococcus* spp, or *Micrococcus* spp) detected in ≥2 blood culture sets within 5 days [15-17]. The date of the first positive blood culture set was regarded as the date of bacteraemia. Polymicrobial bacteraemia was defined as isolation of ≥2 different microorganisms, deemed to represent bacteraemia, within 2 days [18].

Abnormal temperature was defined as >38.0°C or <36.0°C [6]. CRP ≤ 10 mg/L was defined as normal, and CRP > 100 mg/L was considered as highly elevated and has previously been correlated with bacterial infections [19]. SIRS was defined as present if at least two of the following four criteria were fulfilled: body temperature >38.0°C or <36.0°C, respiratory frequency >20 breaths/min or PaCO_2_ < 4,3 kPa, pulse rate >90 beats/min, and leukocyte count >12.0 × 10^9^/L or <4.0 × 10^9^/L [20].

To account for comorbidity, all patients were classified according to the Charlson Comorbidity Score into groups; 0: no comorbidity, 1: light/moderate comorbidity or ≥2: high comorbidity [21].

### Statistical analysis

Patient baseline characteristics were categorised and presented as numbers and percentages. Patients were included at their first MED contact within the study period. The hypothesis of equal proportions was tested using the chi-squared test. ROC for temperature, CRP and SIRS were presented with the area under the operating curve (AUC). For evaluation of the optimal cut-point the following ranges were analysed; Temperature: 35°C–41°C, CRP: 10–350 mg/L. SIRS was analysed by the presence of 0–4 positive SIRS criteria. The optimum cut-points were identified at the receiving operating curve where the Youden Index was maximal, hence where the sum of sensitivity and specificity was the highest [22]. Sensitivity, specificity, predictive positive value, predictive negative value and likelihood ratios were estimated based on all included patients. The 95% confidence intervals were calculated based on a normal distribution of the estimates. Statistical tests were two-sided and a p-value of <0.05 was considered statistically significant. In the case of missing data, the values were registered as normal. Statistical analyses were performed and analysed using Stata 12.1 (Stata Corp LP, College Station, Texas).

### Ethics

The study was approved by the Danish Data Protection Agency (No. 2008-58-0035) and the Danish National Board of Health (No. 3-3013-35). In observational studies review by an Ethics Board is not required according to Danish law.

## Results

During the study period 12,027 patients had a total of 17,332 admissions to the MED. We excluded 39 patients because of missing identification data and included 11,988 patients. Of these 5,492 (45.8%) were males, and the median age was 66 years (range 15–103). Table 1 summarises the baseline characteristics for all included patients.

**Table 1:** Patient characteristics

**Characteristics**		**Blood cultured**		
	**Total n = 11.988**	Not blood-cultured n = 6.489*	Non-bacteraemic patients n = 5.081*	Bacteraemic patients n = 418*
Age, median 66 years (range 15–103)				
- 15–64	5799	3442 (59.4)	2205 (38.0)	152 (2.6)
- 65–79	2974	1447 (48.7)	1384 (46.5)	143 (4.8)
- ≥ 80	3215	1600 (49.8)	1492 (46.4)	123 (3.8)
Sex				
- Male	5492	2861 (52.1)	2396 (43.6)	235 (4.3)
- Female	6496	3628 (55.8)	2685 (41.4)	183 (2.8)
Body temperature > 38.0 or < 36.0 C°	2479	336 (13.6)	1892 (76.3)	251 (10.1)
Heart rate ≥ 90 bpm	4530	1807 (39.9)	2473 (54.6)	250 (5.5)
Respiratory freq ≥ 20 breaths/minute	2750	848 (30.8)	1704 (62.0)	198 (7.2)
Bloodpressure ≤ 90 mmHg	322	119 (36.9)	159 (49.4)	44 (13.7)
Oxygen saturation ≤ 90%	565	177 (31.3)	349 (61.8)	39 (6.9)
PaO_2_ ≤ 10.0 kPa	1883	593 (31.5)	1164 (61.8)	126 (6.7)
Leukocyte count ≥ 12.0 or ≤ 4.0 × 10^9^/L	3904	1370 (35.1)	2285 (58.5)	249 (6.4)
Neutrophil count ≥7.0 × 10^9^/L or ≤ 2.0 × 10^9^/L	6286	2633 (41.9)	3319 (52.8)	334 (5.3)
CRP ≥ 10 mg/dL	6596	2218 (33.6)	3990 (60.5)	388 (5.9)
CRP ≥ 100 mg/dL	2341	331 (14.2)	1733 (74.0)	277 (11.8)
Lactate ≥ 2.0 mmol/L	765	282 (36.9)	422 (55.1)	61 (8.0)
pH ≤ 7.35	482	173 (35.9)	289 (60.0)	20 (4.1)
Creatinine ≥ 177 μmol/L	868	369 (42.5)	424 (48.9)	75 (8.6)
SIRS				
No SIRS	8141	5518 (72.0)	2506 (32.7)	117 (1.5)
SIRS	3847	971 (25.3)	2575 (66.9)	301 (7.8)
Charlson Comorbidity Score				
- 0	5510	3252 (59.0)	2106 (38.2)	152 (2.8)
- 1	2649	1423 (53.7)	1135 (42.9)	91 (3.4)
- ≥2	3829	1814 (47.4)	1840 (48.0)	175 (4.6)

Baseline characteristics of all adult first-time admissions to the Medical Emergency Department. Data based on initial vital values and standard blood samples. CRP: C-reactive protein. SIRS: Systemic Inflammatory Response Syndrome. *The percent is presented horizontally, and can be interpreted as the positive predictive value for the specific variable among the bacteraemic patients.

In total 5,499 (45.9%) patients had blood cultures performed, of which 418 (7.6%) were diagnosed as having true bacteraemia, corresponding to 3.5% of all MED patients.

The most frequent pathogen was *Escherichia coli,* which accounted for 30.4% of all bacteraemia, followed by *Streptococcus pneumoniae* (13.0%) and *Staphylococcus aureus* (10.3%). Polymicrobial bacteraemia accounted for 7.2%. *E. coli* and *S. pneumoniae* represented the highest proportions of bacteria, among the febrile patients and were more likely to have a CRP response >100 mg/L and to have SIRS. Additionally, we found that *E. coli* and *S. aureus* represented the highest proportions among the non-febrile bacteraemic patients (Table 2).

**Table 2:** Pathogens stratified by temperature, CRP and SIRS at arrival to the MED

	**Non-febrile *n = 130 (%)***	**Febrile *n = 251 (%)***	**CRP < 100 *n = 134 (%)***	**CRP ≥ 100 *n = 277 (%)***	**No SIRS *n = 117 (%)***	**SIRS *n = 301 (%)***
*Escherichia coli*	39 (30.0)	78 (31.1)	36 (26.9)	88 (31.8)	32 (27.3)	95 (31.6)
*Klebsiella* species	9 (6.9)	14 (5.6)	9 (6.7)	16 (5.8)	4 (3.4)	21 (7.0)
*Salmonella* species	0 (0.0)	5 (2.0)	4 (3.0)	2 (0.7)	1 (0.8)	5 (1.7)
Other *Enterobacteriaceae*	3 (2.3)	11 (4.4)	6 (4.5)	10 (3.6)	6 (5.1)	11 (3.7)
Anaerobic Gram-negative rods	7 (5.4)	4 (1.6)	2 (1.5)	9 (3.2)	4 (3.4)	6 (2.0)
*Neisseria meningitidis*	2 (1.5)	1 (0.4)	1 (0.7)	2 (0.7)	1 (0.8)	2 (0.7)
Other Gram-negative	11 (8.5)	11 (4.4)	9 (6.7)	13 (4.7)	6 (5.1)	16 (5.3)
*Staphylococcus aureus*	19 (14.6)	21 (8.4)	11 (8.2)	32 (11.5)	13 (11.2)	30 (10.0)
Coagulase-negative staphylococci	7 (5.3)	11 (4.4)	15 (11.2)	9 (3.2)	14 (12.0)	10 (3.3)
*Enterococcus* species	4 (3.1)	7 (2.8)	7 (5.2)	7 (2.5)	8 (6.8)	10 (3.3)
Hemolytic streptococci	4 (3.1)	18 (7.2)	10 (7.5)	13 (4.7)	3 (2.6)	20 (6.6)
Non-hemolytic streptococci	4 (3.1)	7 (2.8)	7 (5.2)	5 (1.8)	6 (5.1)	4 (1.2)
*Streptococcus pneumoniae*	13 (10.0)	38 (15.1)	6 (4.5)	46 (16.6)	10 (8.7)	44 (14.6)
Gram-positive rods	2 (1.5)	3 (1.2)	2 (1.5)	4 (1.4)	3 (2.6)	3 (1.0)
Polymicrobial	6 (4.6)	22 (8.8)	9 (6.7)	21 (7.6)	6 (5.1)	24 (8.0)
Chi Squared Test		<0.001		<0.001		<0.001

Microorganisms detected in 418 *positive* blood cultures defining true community-acquired bacteraemia, obtained within two days after admission in the Medical Emergency Department. “Febrile” represents patients arriving with a temperature >38.0°C, whereas”non-febrile” represents patients arriving with a temperature between 36.0°–38.0°C. The hypothesis of equal proportions was tested using the chi-squared test for the non-febrile and the febrile patients, the patients with a CRP < 100 and CRP ≥ 100, and finally for the patients with and without SIRS. The percent is presented vertically.

Bacteraemia among MED patients was associated with male sex, higher age, higher Charlson Comorbidity Score, an abnormal body temperature (>38.0°C or <36.0°C), CRP >100 mg/L and SIRS, compared to non-bacteraemic patients (Table 1).

### Temperature

**Figure 1:**
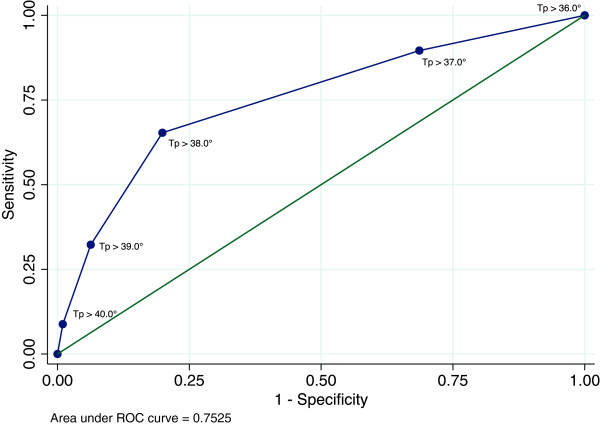
**Receiver operating curve (ROC) for temperature as a diagnostic test for bacteraemia in the medical emergency department.** The analysed cut-points are 36°C–41°C. The figure shows that the ROC for temperature has an area under the curve of 0.75.

In this study 381 of the bacteraemic patients had a temperature measured at arrival, hereof 130/381 (34.1%) were normotherm at arrival to the MED. Figure 1 presents the ROC for different clinical cut-points. The AUC for temperature as a predictor of bacteraemia was 0.75 (95% CI 0.72–0.77), representing a sensitivity of 0.64 and a specificity of 0.81 at a cut-point of >38.0°C. The positive predictive value (PPV) at this cut-point was 11.5% and the negative predictive value (NPV) was 98.3% (Table 3).

**Table 3:** Diagnostic test for CRP, temperature and SIRS

	Non-bacteremic Patients * ***n (%)***	**Bacteremic Patients *n (%)***	**Sensitivity (95% CI)**	**Specificity (95% CI)**	**PPV (95% CI)**	**NPV (95% CI)**	**+ LR (95% CI)**	**- LR (95% CI)**
Temperature > 38.0°C	1879 (88.5)	245 (11.5)	64.3 (59.3–69.1)	80.8 (80.0–81.6)	11.5 (10.2–13.0)	98.3 (98.0–98.6)	3.4 (3.1–3.6)	0.4 (0.4–0.5)
CRP ≥ 100 mg/dL	2064 (88.2)	277 (11.8)	67.4 (62.6–71.9)	79.0 (78.2–79.8)	11.8 (10.6–13.2)	98.3 (98.0–98.6)	3.2 (3.0–3.5)	0.4 (0.3–0.5)
SIRS	3546 (92.2)	301 (7.8)	72.0 (67.4–76.3)	69.4 (68.5–70.2)	7.8 (6.9–8.7)	98.6 (98.3–98.8)	2.4 (2.2–2.5)	0.4 (0.3–0.5)
*Combination test*	7140 (94.7)	397 (5.3)	95.0 (92.4–96.9)	38.3 (37.4–39.2)	5.3 (4.8–5.8)	99.5 (99.3–99-7)	1.5 (1.5–1.6)	0.1 (0.1–0.2)

Results of the diagnostic tests for the clinical parameters temperature, C-reactive protein (CRP), the Systemic Inflammatory Response Syndrome (SIRS) and the *Combination test (Temperature >38°C, or a CRP > 100 mg/dL or ≥2 SIRS criteria).* Analysed in all adult Medical Emergency Department patients (n = 11,988). PPV: Positive Predictive Value. NPV: Negative Predictive Value. +LR: Positive Likelihood Ratio, −LR: Negative Likelihood Ratio. CI: 95% Confidence Interval.

*: Non blood-cultured and blood cultured non-bacteraemic patients. The percent is presented horizontally.

### C-reactive protein

In this study 411 of the bacteraemic patients had a CRP measured at arrival, hereof 134/411 (32.6%) had a CRP < 100 mg/L and 18 (4.4%) bacteraemic patients had a CRP < 10 mg/L.

**Figure 2:**
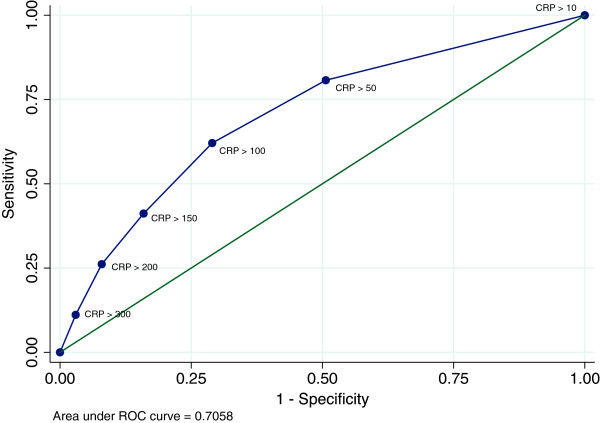
**Receiver operating curve (ROC) for C-reactive protein as a diagnostic test for bacteraemia in the medical emergency department.** The analysed cut-points are CRP 10–350 mg/dL. The figure shows that the ROC for CRP has an area under the curve of 0.70.

Figure 2 presents the ROC for different clinical cut-points. The AUC for CRP as a predictor of bacteraemia was 0.71 (95% CI 0.67–0.73), representing a sensitivity of 0.67 and a specificity of 0.79 at a cut-point of 100 mg/L. The positive predictive value (PPV) at this cut-point of CRP was 11.8% and the negative predictive value (NPV) was 98.3% (Table 3).

### Systemic inflammatory response syndrome

**Figure 3:**
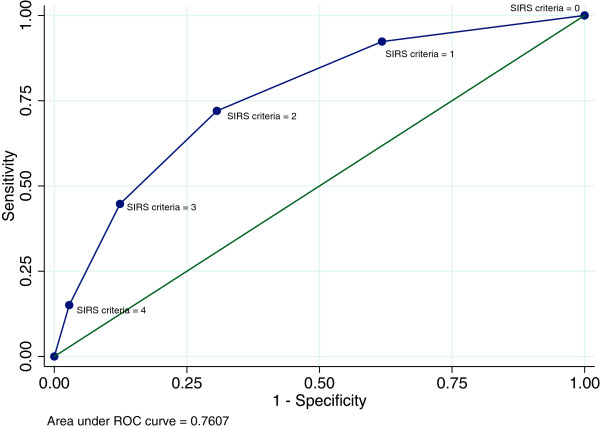
**Receiver operating curve (ROC) for systemic inflammatory response syndrome as a diagnostic test for bacteraemia in the medical emergency department.** The analysed cut-points are SIRS criteria 0–4. The figure shows that the ROC for SIRS has an area under the curve of 0.76.

In this study all bacteremic patients had a SIRS status noted at arrival, hereof 117/418 (28.0%) did not fulfil the criteria for SIRS. Figure 3 presents the ROC for the number of fulfilled criteria for SIRS. The AUC for SIRS as a predictor of bacteraemia was 0.76 (95% CI 0.74–0.78), representing a sensitivity of 0.72 and a specificity of 0.69 at a clinical cut-point of two SIRS criteria. The positive predictive value (PPV) at this cut-point of SIRS was 7.8% and the negative predictive value (NPV) was 98.6% (Table 3).

### Combination

If the three tests were combined, 7,537 (62.8%) of all included patients had either a CRP level ≥100 mg/L, a temperature >38.0°C or fulfilled the criteria for SIRS. Of the 418 bacteraemic patients, 397 (95.0%) fulfilled one or more of these three criteria, representing a diagnostic test for bacteraemia with a sensitivity of 95.0% and a specificity of 38.3%. The positive predictive value (PPV) of this test was 5.3% and the negative predictive value (NPV) was 99.5% (Table 3).

Of the patients who had blood cultures performed in the MED in the present study, 1,287 (23.4%) had a temperature < 38.0°C, a CRP < 100 mg/L, and did not fulfil the criteria for SIRS, and thereby had a low probability of bacteraemia. Of these patients, 21 had bacteraemia, which equals 1.6% of the low risk patients and 5.0% of all bacteraemic patients.

## Discussion

In this population-based study investigating bacteraemia in the MED, we found that 7.6% of all MED patients who had blood cultures drawn, within the first 48 hours after admission, had bacteraemia, corresponding to 3.5% of all MED patients. Although fever, elevated CRP and SIRS all are associated with bacteraemia, they would separately overlook one third of all bacteraemic patients as individual tests. Among all bloodcultured patients admitted to the MED with a low CRP, no SIRS and no fever registered at arrival, only 1.6% had bacteraemia. However, 23% of all blood cultures were drawn among these low risk patients.

Other studies found a positivity rate of 4–12.6% for blood cultures drawn in the ED [1,5,6,9,11,23-25]. In the present study the three most frequently detected pathogens were *E. coli, S. pneumoniae* and *S. aureus*. Similarly, other studies have found *E. coli, S. aureus*, and *Streptococcus* species to be the most frequently detected pathogens in blood cultures drawn in the ED [5,7-9,23,26,27]. We found that among the febrile bacteraemic patients the highest proportion of bacterial species were *E. coli* and *S. pneumoniae*. In addition, *S. pneumoniae* was likely to have a CRP response >100 mg/L and a SIRS response. Except for *E. coli, S. aureus* was most commonly isolated among the non-febrile bacteraemic patients (Table 2). This finding is interesting because it indicates that the lack of fever response may depend on the microorganism present in the blood culture. However, we found no other studies analysing this aspect within the emergency department.

### Single parameters

In this study, a large proportion of the bacteraemic patients presented to the MED without fever. In parallel, other studies have found similar results, ranging between 24–37% [6,11,28,29]. A review by Coburn et al. found that a temperature cut-point of ≥38.5° gave a positive likelihood ratio for bacteraemia of 1.4 [5]. In the present study the positive likelihood ratio for patients with a temperature >38.0 was 3.4 (3.1–3.6). A positive likelihood ratio higher than 10 has previously been proposed as an indication of an acceptable value of a diagnostic test [30]. The combination of a large proportion of bacteraemic patients being non-febrile and the positive likelihood ratio of 3.4 for temperature as a diagnostic test for bacteraemia supports our research hypothesis that the validity of temperature as a single parameter for bacteraemia is only modest.

Many previous studies show that CRP is a difficult biomarker on which to rely solely in diagnosing different kinds of infections, due to the known delay in CRP response [19]. Other studies have evaluated the effect of CRP as a predictor of bacteraemia and have found that CRP has limited validity as a diagnostic test for bacterial infections, because of the low positive predictive value and a poor discriminatory value [7-9,31,32].

Tokuda et al. have shown that the presentation of shaking chills increases the likelihood of bacteraemia (positive likelihood ratio of 4.7) [25]. In the present study we have no systematic information regarding chills, but find in parallel to other studies that a combination of fever with other clinical parameters improves the diagnostic validity.

### Combination of different parameters

A population-based study of all first-time blood cultured patients by Leth et al. has recently proposed SIRS to be an adequate predictor of bacteraemia, and reports a crude odds ratio (OR) for bacteraemia of 7.25 (95% CI 1.75–30.1), and a sensitivity of 96.6%, compared to bloodcultured patients without SIRS [11]. In contrast, the present study finds a sensitivity of SIRS of 64.3%. The discrepancy is probably related to the difference in patient population. Leth et al. studied an inpatient population, which involved both community-acquired and nosocomial bacteraemia. SIRS and temperature equally predicted bacteraemia, despite temperature being one of the SIRS criteria. Temperature had a slightly better specificity while SIRS had a better sensitivity (Table 3).

Although included in the SIRS criteria, we chose to separately analyze temperature as it is readily available, frequently measured and often decisive for the decision to draw blood cultures in daily clinical practice

If the decision to order blood cultures were based only on temperature, CRP or SIRS, (in our study population) one third of all bacteraemic patients would have been overlooked. For the clinician to minimize the risk of overlooking bacteraemic patients, one should use more than one predictor as diagnostic test. At the same time it is necessary to balance the use of resources. Studies on diagnostic strategies have previously focused on identifying low risk patients without the need of a blood culture drawn, thereby reducing healthcare costs without compromising patient care [1].

In the present study 95% of the bacteraemic patients had either a CRP above 100 mg/L, a rectal temperature above 38.0°C, or fulfilled at least two SIRS criteria. In our population it resulted in a negative predictive value of 99.5% (95% CI 99.3-99.7). For unknown reasons 23% of all blood cultures in the present study were performed in the group of patients with low risk of bacteraemia.

Implementation of a combined test, where all patients with either a temperature >38.0°C, a CRP ≥100 mg/L or positive SIRS are blood cultured, presents a very high negative predictive value. This *combined test* would entail a large group (23%) of low risk patients to be withheld from blood cultures, and thereby has the potential to decrease blood cultures drawn in the MED, among patients without suspicion of infection.

Despite the possible decrease in blood cultures among patients in low risk of infection, a combination test would entail a net increase in blood cultures from 45.9% to 63.0% if all patients with either a temperature >38.0°C, a CRP ≥100 mg/L or positive SIRS had blood cultures drawn in the MED. However, if the knowledge of patients in very low risk of bacteraemia is used by the clinician to avoid unnecessary blood cultures, it might be possible to reduce the total number of blood cultures in the MED. But this remains to be confirmed in a prospective controlled trial.

In parallel, Shapiro et al. estimated that by implementing their prediction rule for bacteraemia, blood cultures drawn in the ED, could be reduced by 27%, reflecting a substantial financial saving per year and furthermore, a decreased quantity of false-positive results [1]. In 1990, Bates et al. developed a clinical prediction model that allows the clinician to stratify patients according to their risk of bacteraemia and recommended blood cultures to be taken in all febrile patients (>38.3°C). For patients with a normal temperature and no other risk factors for bacteraemia (as they described) clinicians should consider to withhold blood cultures, which corresponds to this study’s conclusions of determining the risk of bacteraemia based on multiple risk factors and not solely rely on one single parameter [33].

It is a challenge to identify a perfect fast diagnostic test indicating bacteraemia. The right combination of a diagnostic test depends on the basic prevalence of the condition and the associated morbidity or mortality of the disease. Furthermore, it requires a well-validated strategy with high internal as well as external validity and reproducibility across different populations. The present study is not a validated model and cannot serve as such. However, it provides basic information to the clinicians not to rely solely on single parameters such as temperature, CRP or SIRS when they decide to order a blood culture.

The strength of our study is the consecutive inclusion of all adult first-time admission patients arriving to the MED within the study period and the complete follow up on all included patients due to the unique personal identification number used by all Danish citizens.

We are aware of some potential limitations. As patients without blood cultures are classified as negative/non-bacteraemic, this might influence the predictive values for the presented results. However we were not able to take into account, whether or not patients without bloodcultures had undiagnosed bacteraemia. The MED does not receive obvious cardiological, chronic oncological, haematological, nephrological or acute haemorrhagic patients, parturient women and paediatric patients. This means that the results do not apply to all acute medical patients. Furthermore, this is a single-centre study, reflecting the standard care at Odense University Hospital within this period, and therefore the results may not be entirely generalizable to other wards and hospitals.

## Conclusion

34% of the acute medical bacteraemic patients had a normal temperature when arriving at the hospital, 32% had a CRP below 100 mg/L and 28% did not fulfil the criteria for SIRS. However, patients with a normal temperature in combination with CRP < 100 mg/L and no SIRS had a negative likelihood ratio of 0.1 for bacteraemia.

## Competing interests

The authors declare that they have no conflict of interest.

## Authors’ contributions

KPL and ATL conceived the study, designed the trial, and obtained research funding. DPH, SLN and TGJ helped analyze the data; all authors contributed substantially to its revision. KPL takes responsibility for the paper as a whole. All authors read and approved the final manuscript.
